# Impact of insufficient drug efficacy of antiparkinson agents on patient’s quality of life: a cross-sectional study

**DOI:** 10.1186/s12883-015-0360-y

**Published:** 2015-07-05

**Authors:** Jun Tsugawa, Rieko Onozawa, Jiro Fukae, Takayasu Mishima, Shinsuke Fujioka, Yoshio Tsuboi

**Affiliations:** Department of Neurology, Fukuoka University, 7-45-1, Nanakuma, Jonan-ku, Fukuoka, 814-0180 Japan

**Keywords:** Parkinson’s disease, Quality of life, Parkinson’s disease questionnaire-8, Insufficient drug efficacy

## Abstract

**Background:**

To understand the current state of insufficient drug efficacy experienced by patients with Parkinson’s disease (PD) and its effects on quality of life (QOL), we conducted a survey of patients with PD and analyzed the results from 2,630 completed questionnaires.

**Methods:**

The questionnaires inquired about age, sex, Hoehn and Yahr stage, disease duration, drugs currently being taken, and the current state of insufficient drug efficacy; it also included items of the Parkinson’s Disease Questionnaire-8 (PDQ-8). Questionnaires were mailed to members of the Japan Parkinson’s Disease Association.

**Results:**

Approximately 70 % of all subjects reported some type of insufficient drug efficacy, and around half of these experienced this early in the morning or at night. The proportion of subjects who experienced insufficient drug efficacy was found to increase with greater disease severity according to the Hoehn and Yahr stage. However, even among patients with stage I severity, insufficient drug efficacy was reported by approximately 40 % of the respondents. QOL was significantly lower in patients who experienced insufficient drug efficacy than in those who did not (PDQ-8 summary index; 42.0 ± 20.1 vs. 30.0 ± 19.5; *p* < 0.0001).

**Conclusions:**

These results suggest that insufficient drug efficacy might affect the quality of life of patients in most stages PD including the early stages. Therefore, greater awareness of insufficient drug efficacy gained by questioning patients might help medical practitioners in taking appropriate actions.

## Background

Parkinson’s disease (PD), a progressive neurodegenerative disease, has the second highest prevalence in the elderly population after Alzheimer's dementia. It affects approximately 141,000 people in Japan [1], although this number will likely increase further as the Japanese population continues to age rapidly [2]. Recent advances have been made in pharmacotherapies available for PD, such as antiparkinson agents, which mainly include dopaminergic drugs such as levodopa. Dopaminergic medications offer favorable and stable effects in the first few years of administration—called the honeymoon period. However, motor fluctuations such as “wearing-off” and dyskinesia, may develop as complications of long-term treatment of levodopa [3]. It has been reported that such motor complications affect the patient’s quality of life (QOL) to a great degree [4]. Improvements in accuracy of PD diagnosis and treatment have led to prolonged life expectancy in patients with PD, which is nearly identical to that of healthy individuals. Therefore, QOL improvement is now an important issue while planning treatment. Inappropriate pharmacotherapy or insufficient management of wearing-off can lead to insufficient drug efficacy [5]. To investigate the frequency of insufficient drug efficacy during the day and night and the extent to which insufficient drug efficacy impairs QOL, we surveyed patients with PD regarding drug effects and the effect of insufficient drug efficacy on QOL.

## Methods

The protocol used for the study was approved by the Ethics Committee of Fukuoka University Hospital (Fukuoka, Japan). Questionnaires were mailed to 8,001 members (as of August 2012) of the Japan Parkinson’s Disease Association by the secretariat of the association, along with the association’s bulletin. The questionnaire was devised specifically for use in this study. Completed questionnaires and written consent to participate in this study were returned through mail by the respondents. The survey was conducted from August 13 to October 10, 2012. The attributes of patients surveyed in the questionnaire were age, sex, Hoehn and Yahr stage [6], duration of the disease (<3 years, 3–7 years, or ≥7 years), and the names of drugs currently being taken. QOL was evaluated using the Parkinson’s Disease Questionnaire-8 (PDQ-8) [7, 8]. The current state of insufficient drug efficacy was investigated with the following questions: (1) “Does it take time for the drugs to take effect? (yes, no),” (2) “When does the drug take effect during the day? (Early morning, during the day, night; multiple answers possible)” and (3) “What difficulties are caused when insufficient drug efficacy occurs? (Multiple choice, multiple answers possible).”

The results of the survey were then analyzed. The results of whether subjects were troubled by insufficient drug efficacy, at what period(s) they were troubled by insufficient drug efficacy, and what issues were caused by insufficient drug efficacy were documented for all subjects and then classified according to the Hoehn and Yahr stages. For the period(s) when subjects were troubled by insufficient drug efficacy, only responses of “during the day” were counted as “insufficient drug efficacy during the day only” and those of “early morning” and “night” were considered “insufficient drug efficacy during early morning and at night.” Descriptive statistics were calculated for each of the eight items included in the PDQ-8. The PDQ-8 summary index (PDQ-8-SI) was also calculated for each subject, and descriptive statistics for all subjects were evaluated among groups according to the presence or absence of a period of insufficient drug efficacy. The PDQ-8-SI is derived by the sum of PDQ-8 scale scores divided by eight (the number of scales), which yields a score between 0 and 100. In this score, higher the number, more numerous are the health problems [9]. The Student’s *t*-test was used to compare the mean PDQ-8-SI scores according to the presence or absence of a period when the subjects were troubled by insufficient drug efficacy and according to the actual time of the day when they were troubled by insufficient drug efficacy. The level of significance was set at *p* < 0.05 for all tests. We also performed multivariate statistics using the general linear model (GLM) to evaluate whether insufficient drug efficacy influenced PDQ-8-SI after adjustment for several confounding factors (e.g., age, gender, Hoehn and Yahr stage, and duration of the disease). If we found a factor that had a significant interaction with insufficient drug efficacy, we performed a model design that included that interaction term. Gender and Hoehn and Yahr stage were set as the categorical scale, age as the metric scale, and disease duration as the ordinal scale variables of < 3 year = 0, ≧ 3– < 7 year = 1, ≧ 7 = 2.

## Results

Responses were received from 2,632 patients during the survey period. Of these, 2,630 responses were considered valid responses, as two blank questionnaires were excluded. Table 1 lists the attributes of patients. The mean age of the patients was 70.7 years, and women accounted for 54.1 % of the respondents. The most common Hoehn and Yahr stage was stage III (36.5 %), followed by stage IV (12.2 %), stage II (7.9 %), stage V (3.5 %), and stage I (2.9 %). The stage of severity was unclear for 37.0 % of subjects. The most common response for duration of the disease was ≥7 years (63.7 %).

**Table 1:** Patients’ characteristics

Characteristics				
Age, mean ± SD years, (n)	70.7 ± 7.9 (n = 2530)			
Gender, n (%)	Female	:	1424	(54.1 %)
	Male	:	1100	(41.8 %)
	Non-response	:	106	(4.0 %)
Employment status, n (%)	Employed	:	186	(7.1 %)
	Helping with the housework	:	288	(11.0 %)
	Unemployed	:	2095	(79.7 %)
	Non-response	:	61	(2.3 %)
Necessity of caregiving, n (%)	Not requiring caregiving	:	981	(37.3 %)
	Requiring partial caregiving	:	1317	(50.1 %)
	Requiring total caregiving	:	286	(10.9 %)
	Non-response	:	46	(1.7 %)
Type of institutions which the patients attend for treatment, n (%) -multiple answers allowed-	University hospital	:	581	(22.1 %)
	Hospital except for university hospitals	:	1475	(56.1 %)
	Clinic	:	572	(21.7 %)
	Not attending any medical institutions	:	24	(0.9 %)
	Others	:	137	(5.2 %)
	Non-response	:	34	(1.3 %)
Duration of Parkinson’s disease, n (%)	<3 years	:	221	(8.4 %)
	3-7 years	:	681	(25.9 %)
	≥7 years	:	1675	(63.7 %)
	Unknown	:	53	(2.0 %)
Hoehn and Yahr stage, n (%)	Stage I	:	76	(2.9 %)
	Stage II	:	207	(7.9 %)
	Stage III	:	959	(36.5 %)
	Stage IV	:	322	(12.2 %)
	Stage V	:	92	(3.5 %)
	Unknown	:	974	(37.0 %)
Officially acknowledged patient with the specific disease, n (%)	Yes	:	2157	(82.0 %)
	No	:	380	(14.4 %)
	Non-response	:	93	(3.5 %)
Anti-parkinsonian medication, n (%) -multiple answers allowed-	Levodopa	:	1667	(63.4 %)
	Pramipexole IR	:	1076	(40.9 %)
	Pramipexole ER	:	215	(8.2 %)
	Ropinirole	:	474	(18.0 %)
	Cabergoline	:	294	(11.2 %)
	Pergolide	:	294	(11.2 %)
	Bromocriptine	:	58	(2.2 %)
	Entacapone	:	945	(35.9 %)
	Selegiline	:	918	(34.9 %)
	Zonisamide	:	460	(17.5 %)
	Others	:	1427	(54.3 %)
	Non-response	:	166	(6.3 %)

**Fig. 1:**
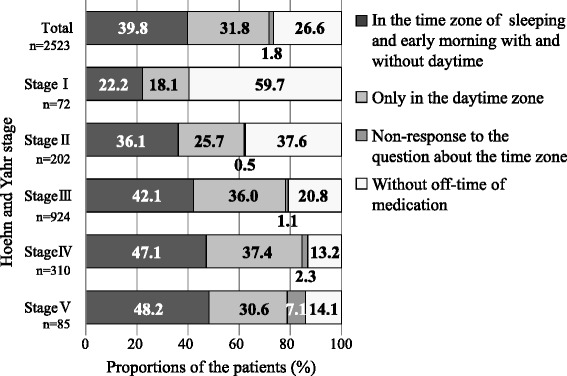
The proportion of patients experienced insufficient efficacy of anti-parkinson medication depend on the time zone stratified by Hoehn &Yahr stages: Data excluding107 patients without response to the question about insufficient efficacy of medication. Data excluding 976 patients with answer as unclear to Hoehn and Yahr stage from stratified analysis

Of the 2,630 respondents who returned completed questionnaires, 73.4 % responded that there were periods when they were troubled by insufficient drug efficacy. According to the Hoehn and Yahr stage, a greater proportion of patients with higher stages of severity responded that there were periods when they were troubled by insufficient drug efficacy. However, the proportion of subjects at stage V was lower than that of subjects at stage IV. With respect to the periods of insufficient drug efficacy, 31.8 % of all subjects responded that they experienced “insufficient drug efficacy during the day only,” while 39.8 % responded that they experienced “insufficient drug efficacy during early morning and at night.” Therefore, more subjects experienced insufficient drug efficacy during early morning and at night than during the day only. However, this trend did not exhibit any correlation with severity of the disease (Fig. 1).

**Fig. 2:**
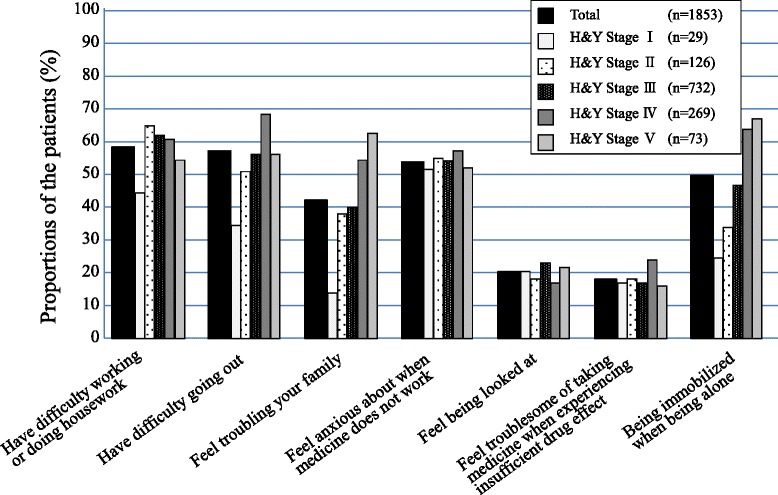
Inconveniences due to experienced insufficient efficacy of anti-parkinson medication: Data excluding 624 patients with answer as unclear to Hoehn and Yahr stage from stratified analysis

The most frequent response to problems caused by insufficient drug efficacy was “cannot work/do housework” (58.7 %), followed in order by “cannot go out” (57.5 %) and “worry about when the drug(s) will stop having effects” (54.4 %). Furthermore, greater severity of the disease led to higher proportions of subjects responding, “cannot go out,” “causing trouble to my family,” and “I lose the ability to move when I am alone” (Fig. 2).

The mean (± standard deviation) PDQ-8-SI was 38.9 ± 20.7, and PDQ-8-SI scores increased with higher Hoehn and Yahr stages. The mean score for subjects who experienced insufficient drug efficacy was significantly higher than the score for those who did not (42.0 ± 20.1 vs. 30.0 ± 19.5; *p* < 0.0001; *t*-test). When divided according to Hoehn and Yahr stages, a significant difference was also observed for subjects with Hoehn and Yahr stages I–III (Table 2). We also found that PDQ-8-SI scores were significantly higher in subjects who experienced “insufficient drug efficacy during early morning and at night” than in those who experienced “insufficient drug efficacy during the day only” (43.3 ± 20.7 vs. 40.7 ± 19.2; *p* = 0.0092; *t*-test) (Table 3). According to Hoehn and Yahr stage, subjects with stages I–II and those who responded that they experienced “insufficient drug efficacy during early morning and at night” tended to have higher scores (Table 3). The highest mean PDQ-8 scores for divided items were for mobility, followed by activities of daily living and emotional well-being (Table 4).

**Table 2:** Comparison of PDQ-8-Summary Index between patient groups with and without insufficient efficacy of medication

	Total		Patients with insufficient efficacy		Patients without insufficient efficacy		*p* value^a^
	n	PDQ-8-SI	n	PDQ-8-SI	Number	PDQ-8-SI	
Total	2422	38.9 ± 20.7	1712	42.0 ± 20.1	625	30.0 ± 19.5	<0.0001
Hoehn &Yahr stages							
Stage I	75	26.7 ± 19.2	29	35.6 ± 22.0	42	19.6 ± 14.5	0.0004
Stage II	194	32.6 ± 20.1	118	38.3 ± 20.6	71	24.3 ± 15.5	<0.0001
Stage III	907	36.8 ± 17.8	697	38.3 ± 17.5	180	31.0 ± 18.2	<0.0001
Stage IV	300	48.0 ± 19.2	251	48.2 ± 19.2	39	45.8 ± 20.0	0.4557
Stage V	77	65.5 ± 23.0	59	67.4 ± 21.5	12	51.3 ± 25.5	0.0246

Data excluding 208 patients without response on PDQ-8-Summary Index and 85 patients without response on experienced insufficient efficacy of medication. Data are expressed as numbers of patients, mean ± SD, or *p* values

^a^:student t-test (comparison between the two groups)

**Table 3:** Patients’ Quality of Life based on the time zone showing insufficient efficacy of anti-parkinsonian medication

	Only in the daytime zone		In the time zone of sleeping and early morning with and without daytime		*p* value^a^
	n	PDQ-8-SI	n	PDQ-8-SI	
	747	40.7 ± 19.2	924	43.4 ± 20.7	0.0092
Hoehn &Yahr stages					
Stage I	13	28.6 ± 20.2	16	41.2 ± 22.4	0.1269
Stage II	49	35.9 ± 19.4	68	40.4 ± 21.4	0.2460
Stage III	319	37.6 ± 17.5	368	38.9 ± 17.4	0.3273
Stage IV	109	46.4 ± 17.6	136	49.9 ± 20.1	0.1460
Stage V	22	63.1 ± 22.0	33	71.3 ± 21.7	0.1761

Data excluding 41 patients without response on the time zones and with insufficient efficacy of medication. Data are expressed as numbers of patients , mean ± SD, or p values

^a^:student t-test (comparison between the two groups)

**Table 4:** Average scores of eight discrete scales in PDQ-8

Category in PDQ-8	(n = 2422)
Mobility	2.1 ± 1.3
Activities of daily living	2.0 ± 1.2
Emotional well being	1.8 ± 1.1
Social support	1.0 ± 1.2
Cognitions	1.5 ± 1.1
Communication	1.4 ± 1.2
Bodily discomfort	1.4 ± 1.2
Stigma	1.1 ± 1.1

Data excluding 208 patients with missing data on PDQ-8. Data are expressed as mean ± SD

The result of a GLM analysis is shown in Table 5. We performed this analysis to evaluate the impact of insufficient drug efficacy on the PDQ-8-SI. We found a significant difference between the interaction term of insufficient drug efficacy + Hohen and Yahr stage and PDQ-8-SI according to the GLM analysis including all interaction terms (P = 0.0218, data not shown). Table 5 presents these interaction terms (insufficient drug efficacy + H & Y stage).

**Table 5:** Multivariate statistical analysis for PDQ-SI(General linear model)

Parameter	B	SE	95 % CI			*p* value
Insufficient drug efficacy						
No	Reference					
Yes	13.12	2.46	8.30	,	17.94	0.000
Hoehn and Yahr stage						
<3	Reference					
>3	8.69	2.26	4.25	,	13.12	0.000
Gender						
Male	Reference					
Female	−2.01	1.01	−3.98	,	−0.03	0.046
Age	0.44	0.07	0.31	,	0.57	0.000
Disease duration	5.47	0.89	3.73	,	7.22	0.000
Interaction						
Insufficient drug efficacy(N), H&Y < 3	Reference					
Insufficient drug efficacy(N), H&Y≧3	Reference					
Insufficient drug efficacy(Y), H&Y < 3	Reference					
Insufficient drug efficacy(Y), H&Y≧3	−5.71	2.81	−11.22	,	−0.19	0.042

*B* regression coefficient; 95 % *CI* 95 % confidence interval; *SI* standard error; *GLM* General linear model; *H&Y* Hoehn and Yahr stage

These analyses confirmed that insufficient drug efficacy has a significant effect on PDQ-8-SI, independent of confounding factors such as age, gender, Hoehn and Yahr stage, and duration of the disease; PDQ-8-SI of insufficient drug efficacy with a “Yes” response was 13.12 (95 % CI:8.30,17.94) higher than that with a “No” response to insufficient drug efficacy (*p* < 0.001). However, we also found a significant interaction between insufficient drug efficacy and Hoehn and Yahr stage; Hoehn and Yahr stage group of ≧ 3 found PDQ-SI increasing score reduced 5.71(95 % CI: 2.81, −11.22) compared to Hoehn and Yahr stage group of <3.

## Discussion

In the present study, approximately 70 % of all patients with PD responded that there were “periods when they were troubled by insufficient drug efficacy.” Moreover, among patients who were classified to be at Hoehn and Yahr stage I, approximately 40 % felt insufficient drug efficacy during the daytime and/or nighttime. Stacy *et al*. [10] investigated the prevalence of wearing-off in patients with PD treated with levodopa for ≤5 years using the Wearing-off Questionnaire-32 (WOQ-32). They found that 57 % of subjects experienced wearing-off, although physicians recognized the presence of wearing-off only in 40 % of patients, indicating a significant deviation from reports by physicians about whether wearing-off was present or not. Wearing-off is generally recognized as a complication in the progressive stage of PD [11]. However, insufficient drug efficacy experienced by subjects in relatively early stages in the present study indicated the possibility of under-medication in which the duration of the effect or dosage was insufficient. These findings suggest the importance of proactively confirming drug efficacy and identifying effects of medical treatment on QOL. Our results also indicate an overall high frequency of insufficient drug effects, including subjects with advanced stages of PD. This could be due to not only an increase in wearing-off but also because patients compared current with past drug efficacy. These results may also be reflective of the patients’ dissatisfaction with their current disease states. In any case, appropriate questioning of patients by physicians is crucial to understand their current state and to offer treatment with suitable medications.

Our survey also revealed that the frequency of responses to items related to motor symptoms, such as “cannot go out” and “I lose the ability to move when I am alone,” increased in patients with higher stages of severity. The item “worry about when the drug(s) will stop having effects” had a high frequency, regardless of the stage of severity, even in subjects with early stages of PD. As this was the most common response to problems caused by insufficient drug efficacy by subjects with Hoehn and Yahr stage I, it appears that patients with early stages of PD require sufficient explanation and psychological support. Furthermore, the highest proportion of subjects who responded that they “cannot work/do housework” occurred among subjects with Hoehn and Yahr stage II. Thereafter, the proportion decreased with greater severity of the disease. These results suggest the need for treatment that takes the social environment of the patient into consideration.

The results of the present study indicated that more than half of patients who responded that there were periods when they were troubled by insufficient drug efficacy replied that they were troubled not only during the day but during early morning and at night as well. These results were the same for subjects with Hoehn and Yahr stage I. Although nighttime and early morning symptoms have conventionally been considered a problem for patients with advanced stages of PD, these symptoms also occur in patients with early stages of PD. Patients who experience insufficient drug efficacy at night or during early morning had significantly higher PDQ-8-SI scores than those who did not. These patients were also likely to have lower QOL. Chapuis *et al.* [12] conducted an evaluation using the PDQ-39 SI and reported that the presence of nighttime and early morning motor complications significantly compromised QOL. In particular, immobility during the night exacerbated all eight dimensions included in the PDQ-39. Havlikova *et al.* [13] reported that sleep disorders affected QOL and insufficient drug efficacy at night and during early morning reduced the amount of good quality sleep, thereby reducing QOL.

This study is based on objective evaluation of the patients; therefore, “insufficient drug efficacy” in this study includes various factors such as under medication causing inadequate dopaminergic stimulation, which affects patients both in the daytime and early morning, and wearing-off phenomenon only affects in the daytime. We should recognize the necessity to resolve these problem in each patient.. It is not clear whether insufficient drug efficacy during the day, night, or early in the morning has the most effect on QOL.

Here, we used the PDQ-8 to evaluate QOL. The PDQ-8 questionnaire is a shortened version of PDQ-39 and has been established as an index to evaluate the QOL of patients with PD [14]. However, no previous used the PDQ-8 to investigate Japanese subjects. In the present study, the mean PDQ-8-SI score was 38.9, which was higher than that reported in a validation study^1^ (mean score, 27.16). The higher score in the present study may have been due to effects of disease severity. Factors affecting QOL include Hoehn and Yahr stage, UPDRS part III score, and duration of the disease [8]. The mean Hoehn and Yahr stage for Japanese patients included in a previous validation study was 2.85 ± 0.95, while the mean severity stage for subjects in the present study was 3.09 ± 0.85, with patients at stage IV or stage V accounting for 15.7 % of all subjects. Scores for patients with Hoehn and Yahr stages II and III were consistent with those reported in a study conducted overseas [15].

In the present study, when PDQ-8-SI scores were assessed according to severity of the disease, we found that QOL was lower in patients who experienced insufficient drug efficacy, regardless of disease severity. In particular, QOL was significantly lower in patients who experienced insufficient drug efficacy and who had Hoehn and Yahr stages I-III. Among patients at stage I, the mean PDQ-8-SI score was 35.6 ± 22.0 for individuals who responded that they experienced insufficient drug efficacy and 19.6 ± 14.5 for those who responded that they did not. According to Jenkinson *et al*. [8], the mean PDQ-8-SI score for patients at stage I was 17.74, suggesting that insufficient drug efficacy may affect QOL. Stacy *et al.* [16] reported that motor complications decreased QOL. However, according to an investigation by Marras *et al.* [17], motor complications did not largely influence the QOL of patients with early stages of PD. There were limitations to the interpretation of the present results because other factors that could affect QOL were not examined. However, when patients with identical disease severity were examined, those who experienced insufficient drug efficacy had significantly lower QOL. This suggests that when prescribing medications, physicians should consider the fact that motor complications such as wearing-off affect QOL even in patients with early stages of PD.

Our results revealed no statistical difference of PDQ-8 SI between patients with and without insufficient efficacy only in patients at the Yahr 4 stage. It is speculated that majority of patients with the Yahr 4 stage would develop the wearing-off effect and be aware of these symptoms disturbing their quality of life (QOL). Therefore, the physician needs to increase the dose of dopaminergic medication including L-DOPA and dopamine agonists. In addition, there is a possibility that non-motor symptoms, which are not resolved with dopaminergic medication, strongly affect the QOL of patients. These non-motor symptoms could decrease the QOL of both patients with and without insufficient drug efficacy.

The questionnaire used in the present study to survey patients may have led to some variation because of individual interpretations by the respondents. Moreover, as there were limits to the items that could be surveyed, it was not possible to assess all factors that affect QOL. Furthermore, because this survey was voluntary, the questionnaire recovery rate was only 32.9 %. However, the results clarified that insufficient drug efficacy occurred at a high frequency from early stages in over 2,600 patients with PD, with no restrictions on age or severity of the disease, and had a detrimental influence on QOL.

This study included few patients with the mild stage of the disease possibly because the support group (Japan Parkinson’s Disease Association) itself included fewer patients in the mild stage of the disease. However, we could not rule out the possibility that fewer patients in the mild stage participated in our study because of lack of motivation to respond to the survey. If this were the case, it could account for the higher frequency of insufficient drug efficacy in the mild PD population included in this study.

The large sample size could definitely lower the chance of random errors; however, because of the study design, one other limitation is the possibility of systematic errors or biases, such as information bias. Therefore, further studies should be performed to validate our findings.

## Conclusions

The results of the present study indicated that nearly 70 % of all our patients with PD and approximately 40 % of those with Hoehn and Yahr stage I experienced insufficient drug efficacy. The results also indicated that more than half of these patients experienced insufficient drug efficacy not only during the day but also at night and in the early morning. QOL was significantly decreased in patients who responded that they were troubled by insufficient drug efficacy. Therefore, physicians should question patients regarding insufficient drug efficacy at night and during early morning. Further studies on this topic are needed to help facilitate better QOL in patients with PD.

## Abbreviations

PD: Parkinson’s disease
QOL: Quality of life
PDQ-8: Parkinson’s Disease Questionnaire-8
PDQ-8-SI: PDQ-8 summary index
WOQ-32: Wearing-off Questionnaire-32
